# Supplementary material for: scMoMtF: An interpretable multitask learning framework for single-cell multi-omics data analysis
Source: PLoS Comput Biol. 2024 Dec 18;20(12):e1012679. doi: 10.1371/journal.pcbi.1012679 (PMC11654984; doi:10.1371/journal.pcbi.1012679)
Supplement: Attachment — Submitted filename: response.pdf [file pcbi.1012679.s001.pdf]

Authors' Responses to the Editor's Comments
On the paper

"scMoMtF: An Interpretable Multitask Learning Framework for Single-Cell Multi-omics Data Analysis"

By Wei Lan, Tongsheng Ling, Qingfeng Chen, Ruiqing Zheng, Min Li, Yi Pan

Dear the editor and reviewers,

We would like to thank you for your careful reading, helpful comments, and constructive suggestions, which have significantly improved the presentation of the paper. We have carefully read all comments and revised our manuscript based on them. Furthermore, the language of this paper has also been checked by a native speaker. In the following, we give our responses to the review comments. We copied the original comments from the reviewers, and made them italic. Our responses were given under each comment. We wish our revised manuscript and responses would satisfy you.

Yours Sincerely,

Wei Lan, Tongsheng Ling, Qingfeng Chen, Ruiqing Zheng, Min Li, Yi Pan

Answers to Reviewer 1

1. The authors should ensure that all terms used in the paper are presented with their full names upon first mention. For instance, terms like SHARE-seq should be fully defined to ensure clarity for readers who may not be familiar with the abbreviations.

Author's Response: Thanks for your point. In the revised version, we double-check the content of the paper and provide the full name of abbreviation:

"For example, single-nucleus chromatin accessibility and mRNA expression sequencing (SNARE-seq) [9] and simultaneous high-throughput ATAC and RNA expression with sequencing (SHARE-seq) [10] techniques can measure gene expression and chromatin accessibility simultaneously in the same cell.

…………..

We evaluate the dimension reduction performance of scMoMtF on four different datasets from SNARE-seq, peripheral blood mononuclear cell (PBMC) [25], SHARE-seq and CITE-seq [26].

…………..

We visualize the dimension reduction data of each model by using uniform manifold approximation and projection (UMAP) (Fig. 2a-d)."

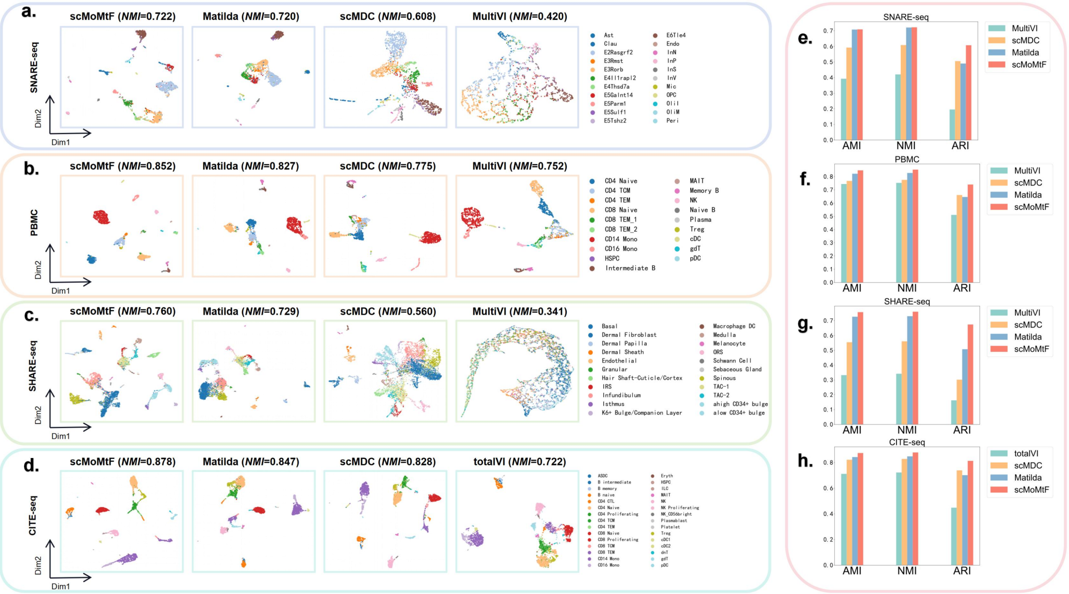

Fig 2. Visualization and performance evaluation of dimension reduction task of scMoMtF compared with other comparison algorithms. a-c Visualization of dimension reduction data generated by scMoMtF, Matilda, scMDC, and MultiVI on SNARE-seq, PBMC, and SHARE-seq datasets. d Visualization of dimension reduction data generated by scMoMtF, Matilda, scMDC and totalVI on the CITE-seq dataset. e-g Evaluate the clustering performance of dimension reduction data generated by scMoMtF, Matilda, scMDC, and MultiVI on SNARE-seq, PBMC, and SHARE-seq datasets using AMI, NMI, and ARI. h The clustering performance of dimension reduction

data generated by scMoMtF, Matilda, scMDC and totalVI on CITE-seq dataset.

References:

[9] Chen S, Lake BB, Zhang K. High-throughput sequencing of the transcriptome and chromatin accessibility in the same cell. Nature biotechnology. 2019;37(12):1452–1457.

[10] Ma S, Zhang B, LaFave LM, Earl AS, Chiang Z, Hu Y, et al. Chromatin potential identified by shared single-cell profiling of RNA and chromatin. Cell. 2020;183(4):1103–1116.

[25] PBMC from a healthy donor - granulocytes removed through cell sorting (10k), Singl e Cell Multiome ATAC + Gene Exp Dataset by Cell Ranger ARC 1.0.0, 10x Genomics; 2 020. Available from: https://support.10xgenomics.com/single-cell-multiome-atac-gex/ datasets/ 1.0.0/pbmc_granulocyte_sorted_10k.

[26] Hao Y, Hao S, Andersen-Nissen E, Mauck WM, Zheng S, Butler A, et al. Integrated analysis of multimodal single-cell data. Cell. 2021;184(13):3573–3587.

2. In the figures, the first letters of words should be capitalized for consistency and professionalism. For example, in Figure 7e, ensure that all labels adhere to this formatting rule.

Author's Response: Thanks for your point. In revised version, we capitalize the first letters of all figures. In addition, we use table to replace the Figure 7e for representing running time with more intuitive way:

"In the field of single-cell multi-omics data analysis, the performance and training efficiency of deep learning models are important criteria for evaluating their superiority. We record the runtime of all models in the experiment and the results are shown in Table 1. It can be found that scMoMtF has a significantly shorter training time by compared to other models (including both multi-task and single-task models). Although the training time of scmap [32] is shorter than scMoMtF, the accuracy of scmap in the cell classification task is much lower than scMoMtF. Therefore, scMoMtF not only demonstrates superior performance in multitasking capabilities but also exhibits exceptional competitiveness in training efficiency. And scMoMtF is a powerful tool for efficiently handling single-cell multi-omics data."

Table 1. Task training time (in seconds) of each method on different datasets.

Task	Method	SNARE-seq (9190 Cells)	PBMC (9631 Cells)	SHARE-seq (17115 Cells)	CITE-seq (32231 Cells)
Dimension Reduction	MultiVI	613	782	2269	-
	totalVI	-	-	-	2039
	scMDC	313	324	417	1033
Cell Classification	scPred	382	305	1260	4106
	scClassify	29	37	157	106
	scmap	3	5	15	9

	CHETAH	39	22	81	84
Data Simulation	SPARSim	152	180	279	587
	Matilda	40	42	67	143
Multiple Tasks	scMoMtF	14	14	28	46

Note: Among all the models scMoMtF and Matilda are multi-task models and the rest are single-task models. - : indicates that the model cannot be applied to the dataset.

References:

[32] Kiselev VY, Yiu A, Hemberg M. scmap: projection of single-cell RNA-seq data across data sets. Nature methods. 2018;15(5):359–362.

3. The process of calculating the indicators used in the paper, such as Adjusted Rand Index (ARI) and Normalized Mutual Information (NMI), should be explicitly shown. Providing a detailed explanation of how these indicators are computed will help readers understand the methodology and validate the results. It is recommended that the authors be able to add relevant content to ensure the reproducibility of the study.

Author's Response: Thanks for your point. In the revised version, we provide a detailed description of the calculation process for the Adjusted Rand Index (ARI) and Normalized Mutual Information (NMI):

"Evaluation metrics

Adjusted Rand Index (ARI). The ARI score measures measures the agreements between two sets P (the clustering result of the predicted by model) and T (the clustering result of real label). Assuming N_1 represent the number of pairs of objects that are assigned to the same cluster in both P and T; N_2 represent the number of pairs of objects that are assigned to different clusters in both P and T; N_3 represent the number of pairs of objects that are assigned to the same cluster in P but to different clusters in T; N_4 represent the number of pairs of objects that are assigned to the same cluster in T but to different clusters in P. the ARI is calculated using the following formula:

$$ARI = \frac{\binom{n}{2}(N_1+N_2)-[(N_1+N_3)(N_1+N_4)+(N_4+N_2)(N_3+N_2)]}{\binom{n}{2}-[(N_1+N_3)(N_1+N_4)+(N_4+N_2)(N_3+N_2)]} \tag{13}$$

And the ARI is near one when the clustering result from the model aligns well with the observed cell type labels, while it is close to zero when the clustering resembles a random assignment.

Normalized mutual information (NMI). Similar to ARI score, let $P = \{P_1, P_2, ..., P_{np}\}$ and $T = \{T_1, T_2, ..., T_{nt}\}$ be the predicted and real labels on a dataset with n cells. NMI is defined as follows:

$$NMI = \frac{I(P,T)}{\max\{H(P),H(T)\}} \tag{14}$$

$$I(P,T) = \sum_{i=1}^{np} \sum_{j=1}^{nt} |P_i \cap T_j| \log \frac{n|P_i \cap T_j|}{|P_i| \times |T_j|} \quad (15)$$

$$H(P) = -\sum_{i=1}^{np} |P_i| \log \frac{|P_i|}{n} \quad (16)$$

$$H(T) = -\sum_{j=1}^{nt} |T_j| \log \frac{|T_j|}{n} \quad (17)$$

where $I(P,T)$ represents the mutual information between P and T, $H(\mathrm{P})$ and $H(\mathrm{T})$ are the entropy of partitions.

Adjusted Mutual Information (AMI). AMI is an adjusted version of NMI and AMI takes into account the effects of random assignment and category imbalance. AMI is defined as follows:

$$AMI(P,T) = \frac{I(P,T) - E\{I(P,T)\}}{\max\{H(P), H(T)\} - E\{I(P,T)\}} \quad (18)$$

where $E\{I(P,T)\}$ is the expected mutual information between P and T under random labeling assumption."

4. The details of how the concatenate operation in Equation 3 is realized should be thoroughly explained. A comprehensive description of this process will aid in the understanding of the algorithm 's implementation. Ensuring that every step of the methodology is well-documented is essential for readers who wish to replicate or build upon this work.

Author's Response: Thanks for your point. In the revision, we explain the connection operation in Equation 3 with detail:

"Next, $h_i^{(1)}$ and $h_i^{(2)}$ are concatenated to input into the cell encoder E_{Cell} to obtain the final cell embedding z_i of cell i:

$$z_i = E_{Cell}\left(concatenate\left(h_i^{(1)}, h_i^{(2)}\right)\right) \quad (3)$$

where the length of $h_i^{(1)}$ and $h_i^{(2)}$ are $l_i^{(1)}$ and $l_i^{(2)}$, respectively. And the length of concatenated embedding is $l_i^{(1)} + l_i^{(2)}$."

5. Could the authors describe the advantages and disadvantages of the method in more detail in the discussion section and describe the future directions for improvement.

Author's Response: Thanks for your point. In the revision, we add a detailed discussion of the advantages and disadvantages of our method. Additionally, we also discuss the future work:

"The current single-cell sequencing technology can simultaneously measure multiple molecular information (RNA, chromatin accessibility and proteins) of the same cell. It demands to combine different tasks to fully understand these single-cell multi-omics data. However, many current methods for analyzing single-cell multi-omics data are designed to perform a single task

and rely on specific datasets which make it fail to fully utilize the potential of single-cell multi-omics data. For example, scMDC performs well on PBMC and CITE-seq datasets but performs poorly on other datasets in dimension reduction task. And the accuracy of scmap is significantly lower on the SHARE-seq dataset in cell classification task. In addition, many methods lack corresponding interpretability which is difficult to provide biologically reliable insights. To address this issue, we propose an interpretable multitask framework (scMoMtF) for comprehensive analyzing single-cell multi-omics data. We evaluate the performance of scMoMtF in data dimension reduction, cell classification and data simulation tasks. The experimental results indicate that scMoMtF can obtain better performance on all tasks and correct the batch effect of single-cell multi-omics data. In addition, scMoMtF can reveal potential marker genes to provide reliable biological insights. Furthermore, scMoMtF can be a convenient analysis tool without too much parameters adjustment and training time.

In future work, we also plan to explore potential improvements to the method, such as enhancing its computational efficiency to handle larger datasets more effectively and expanding its applicability to a broader range of single-cell multi-omics datasets. Moreover, we will investigate potential applications of scMoMtF in related areas, such as integrating spatial transcriptomics data or applying the framework to other types of multi-modal data."

Answers to Reviewer 2

1. As the authors mentioned, the model "during the training process of dimension reduction and cell classification tasks, the interpretability module is used to enhance this process." Could you explain in more detail what you mean by this statement.

Author's Response: Thanks for your point. In our revised version, we provide a clearer explanation of interpretability module:

"In addition, the interpretability module is used to provide additional insights on the importance of genes in dimension reduction task and cell classification task. This helps to discover potential marker genes in the cell (Fig. 1c)."

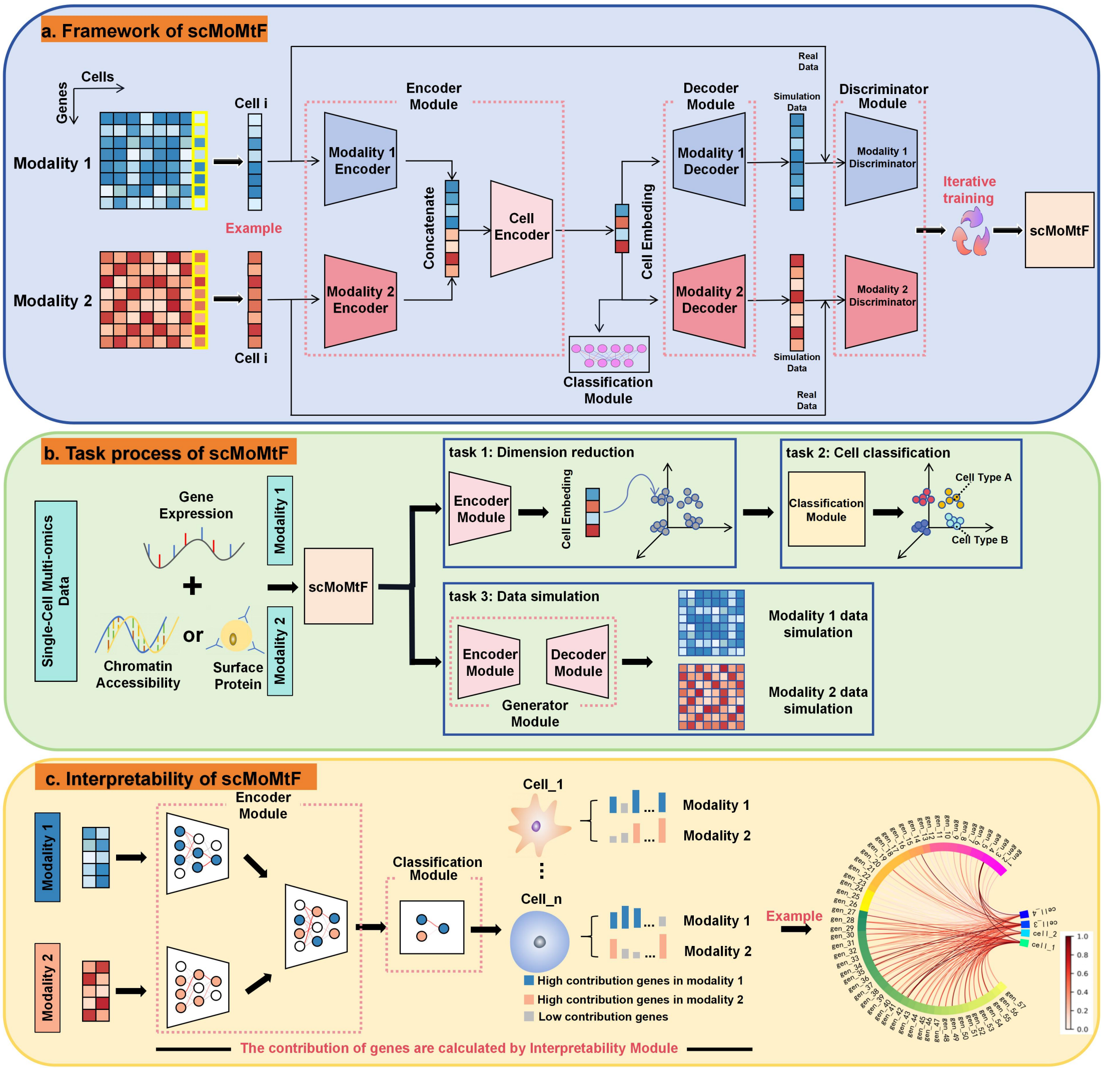

Fig 1. scMoMtF overall structure and task module diagram. a scMoMtF uses the matched single-cell multi-omics data as the input to the model and the overall model framework is encoder-decoder-discriminator-classifier. b The tasks process of scMoMtF. c The research process for the interpretability of scMoMtF.

2. *In the dimension reduction task, the authors use the clustering results of the k-means method for*

the corresponding metrics computation and the corresponding parameters of the method should be given for the reader's reproduction.

Author's Response: Thanks for your point. In our revised version, we add the corresponding details of k-means method:

"In order to intuitively show the dimension reduction performance of each method, we use k-means clustering algorithm to cluster the cell embedding with same parameters (n clusters is the number of cell types for the corresponding dataset and n init is set to 30)."

3. The process of calculating the indicators used in the paper, such as Adjusted Rand Index (ARI) and Normalized Mutual Information (NMI), should be explicitly shown. Providing a detailed explanation of how these indicators are computed will help readers understand the methodology and validate the results. It is recommended that the authors be able to add relevant content to ensure the reproducibility of the study.

Author's Response: Thanks for your suggestion. In our revised version, we add a "Evaluation metrics" section with detailed description of the experimental evaluation metrics in the paper.

"Evaluation metrics

Adjusted Rand Index (ARI). The ARI score measures measures the agreements between two sets P (the clustering result of the predicted by model) and T (the clustering result of real label). Assuming N_1 represent the number of pairs of objects that are assigned to the same cluster in both P and T; N_2 represent the number of pairs of objects that are assigned to different clusters in both P and T; N_3 represent the number of pairs of objects that are assigned to the same cluster in P but to different clusters in T; N_4 represent the number of pairs of objects that are assigned to the same cluster in T but to different clusters in P. the ARI is calculated using the following formula:

$$ARI = \frac{\binom{n}{2}(N_1+N_2)-[(N_1+N_3)(N_1+N_4)+(N_4+N_2)(N_3+N_2)]}{\binom{n}{2}-[(N_1+N_3)(N_1+N_4)+(N_4+N_2)(N_3+N_2)]} \quad (13)$$

And the ARI is near one when the clustering result from the model aligns well with the observed cell type labels, while it is close to zero when the clustering resembles a random assignment.

Normalized mutual information (NMI). Similar to ARI score, let $P = \{P_1, P_2, ..., P_{np}\}$ and $T = \{T_1, T_2, ..., T_{nt}\}$ be the predicted and real labels on a dataset with n cells. NMI is defined as follows:

$$NMI = \frac{I(P,T)}{\max\{H(P),H(T)\}} \quad (14)$$

$$I(P,T) = \sum_{i=1}^{np}\sum_{j=1}^{nt} |P_i \cap T_j| \log\frac{n|P_i \cap T_j|}{|P_i| \times |T_j|} \quad (15)$$

$$H(P) =- \sum_{i=1}^{np} |P_i| \log\frac{|P_i|}{n} \quad (16)$$

$$H(T) = -\sum_{j=1}^{nt} |T_j| \log \frac{|T_j|}{n} \quad (17)$$

where $I(P,T)$ represents the mutual information between P and T, $H(\mathrm{P})$ and $H(\mathrm{T})$ are the entropy of partitions.

Adjusted Mutual Information (AMI). AMI is an adjusted version of NMI and AMI takes into account the effects of random assignment and category imbalance. AMI is defined as follows:

$$AMI(P,T) = \frac{I(P,T) - E\{I(P,T)\}}{\max\{H(P),H(T)\} - E\{I(P,T)\}} \quad (18)$$

where $E\{I(P,T)\}$ is the expected mutual information between P and T under random labeling assumption."

4. In the comparison experiments of the training efficiency of each model, could you show the training time of all the comparison experiments mentioned in the paper. This can visualize the advantages of the authors' model more.

Author's Response: Thanks for your suggestion. In our revised version, we calculate the training time of all the models in the experiment to more intuitively demonstrate the advantages of our model in training efficiency:

"In the field of single-cell multi-omics data analysis, the performance and training efficiency of deep learning models are important criteria for evaluating their superiority. We record the runtime of all models in the experiment and the results are shown in Table 1. It can be found that scMoMtF has a significantly shorter training time by compared to other models (including both multi-task and single-task models). Although the training time of scmap [32] is shorter than scMoMtF, the accuracy of scmap in the cell classification task is much lower than scMoMtF. Therefore, scMoMtF not only demonstrates superior performance in multitasking capabilities but also exhibits exceptional competitiveness in training efficiency.And scMoMtF is a powerful tool for efficiently handling single-cell multi-omics data."

Table 1. Task training time (in seconds) of each method on different datasets.

Task	Method	SNARE-seq (9190 Cells)	PBMC (9631 Cells)	SHARE-seq (17115 Cells)	CITE-seq (32231 Cells)
Dimension Reduction	MultiVI	613	782	2269	-
	totalVI	-	-	-	2039
	scMDC	313	324	417	1033
Cell Classification	scPred	382	305	1260	4106
	scClassify	29	37	157	106
	scmap	3	5	15	9
	CHETAH	39	22	81	84
Data Simulation	SPARSim	152	180	279	587

	Matilda	40	42	67	143
Multiple Tasks	scMoMtF	14	14	28	46

Note: Among all the models scMoMtF and Matilda are multi-task models and the rest are single-task models. - : indicates that the model cannot be applied to the dataset.

References:

[32] Kiselev VY, Yiu A, Hemberg M. scmap: projection of single-cell RNA-seq data across data sets. Nature methods. 2018;15(5):359–362.

5. There should be consistency in the descriptions in the paper; the authors give a complete description of the CITE-seq and ADT techniques, but not the SNARE-seq and SHARE-seq techniques. It is hoped that the authors will take note of such errors and correct them.

Author's Response: Thanks for your point. In the revised version, we provide the full name of "SNARE-seq" and "SHARE-seq":

"For example, single-nucleus chromatin accessibility and mRNA expression sequencing (SNARE-seq) [9] and simultaneous high-throughput ATAC and RNA expression with sequencing (SHARE-seq) [10] techniques can measure gene expression and chromatin accessibility simultaneously in the same cell."

References:

[9] Chen S, Lake BB, Zhang K. High-throughput sequencing of the transcriptome and chromatin accessibility in the same cell. Nature biotechnology. 2019;37(12):1452–1457.

[10] Ma S, Zhang B, LaFave LM, Earl AS, Chiang Z, Hu Y, et al. Chromatin potential identified by shared single-cell profiling of RNA and chromatin. Cell. 2020;183(4):1103–1116.

Answers to Reviewer 3

1. In methodology, the method is to model two modalities. How if the data has more than two omics datasets?

Author's Response: Thanks for your suggestion. Our model is designed for single-cell multi-omics data with two modalities. If the datasets include more than two omics, the model is easy to extend to handle multiple modalities as the follow:

The model can be achieved by modifying the architecture to accept multiple inputs. For example, if the dataset has three omics data (e.g., RNA, chromatin accessibility and surface protein), we can increase the number of the modality encoders to three in the model. And the information vector of each omics data is extracted by the modality encoder and then concatenated. Finally, the connected vector is sent into the cell encoder to obtain the final embedding vector and perform downstream tasks.

2. No details about how the developed method scMoMtF are benchmarked to other methods. The method is benchmarked to multiple methods in each aspect (dimension reduction or cell classification or batch effect correction etc.) But there is a lack of description or introduction of each method. For example, no description of the method that was benchmarked to like SHAP (Page7, Line174).

Author's Response: Thanks for your suggestion. In our revised manuscript, we add descriptions of all the methods in the experiment:

"Dimension reduction methods

MultiVI (https://github.com/scverse/scvi-tools). The input of MultiVI are matched raw count matrices of RNA and gene activity score matrices from ATAC. We use the default parameters provided by the author in the experiment. Following the author's tutorial, we first connect the RNA and ATAC data and then train the model through the 'scvi.model.MULTIVI.setup anndata', 'scvi.model.MULTIVI' and 'train' functions. The final embedding can be obtained by the 'get latent representation' function.

totalVI (https://github.com/scverse/scvi-tools). The input of totalVI are matched raw count matrices of RNA and ADT. We use the default parameters provided by the author in the experiment. Following the author's tutorial, we normalize the raw data through the 'normalize total' and 'log1p' functions. And then we train the model through the 'scvi.model.TOTALVI.setup anndata', 'scvi.model.TOTALVI' and 'train' functions. The final embedding can be obtained by the 'get latent representation' function.

scMDC (https://github.com/xianglin226/scMDC). There are two types inputs of scMDC: matched raw count matrices of RNA and gene activity score matrices from ATAC; matched raw count matrices of RNA and ADT. We use the default parameters provided by the author in the experiment. Following the author's tutorial, we normalize the raw data through the 'normalize' function. And then we train the model through the 'scMultiCluster' and 'pretrain autoencoder' functions. The final embedding can be obtained by the 'encodeBatch' function.

Matilda (https://github.com/PYangLab/Matilda). There are two types inputs of Matilda: matched raw count matrices of RNA and gene activity score matrices from ATAC; matched raw count matrices of RNA and ADT. We use the default parameters provided by the author in the experiment. Following the author's tutorial, we normalize the raw data through the 'compute_log2' and 'compute_zscore' functions. Then we concatenate the data of the two modalities and train the model through the 'CiteAutoencoder_SHAREseq' (or 'CiteAutoencoder_CITEseq') and 'train_model' functions. The final embedding can be obtained by the 'get_encodings' function.

Cell classification methods

scPred (https://github.com/powellgenomicslab/scPred). The input of scPred is raw count matrices of RNA. We use the default parameters provided by the author in the experiment. Following the author's tutorial, we preprocess the raw data through the 'NormalizeData', 'FindVariableFeatures', 'ScaleData', 'RunPCA' and 'RunUMAP' functions. And then we train the model through the 'getFeatureSpace'and 'trainModel' functions. The result of cell classification can be obtained by the 'scPredict' function.

scClassify (https://github.com/SydneyBioX/scClassify). The input of scClassify is raw count matrices of RNA. We use the default parameters provided by the author in the experiment. Following the author's tutorial, we normalize the raw data through the 'NormalizeData' function. And then we train the model and obtain the result of cell classification through the 'scClassify' function.

scmap (https://github.com/hemberg-lab/scmap). The input of scmap is raw count matrices of RNA. We use the default parameters provided by the author in the experiment. Following the author's tutorial, we train the model and obtain the result of cell classification through the 'selectFeatures', 'indexCluster' and 'scmapCluster' functions.

CHETAH (https://github.com/jdekanter/CHETAH). The input of CHETAH is raw count matrices

of RNA. We use the default parameters provided by the author in the experiment. Following the author's tutorial, we train the model and obtain the result of cell classification through the 'CHETAHclassifier' function.

Data simulation methods

SPARsim (https://gitlab.com/sysbiobig/sparsim). The input of SPARsim is raw count matrices of RNA. Following the author's tutorial, we normalize the raw data through the 'scran_normalization' function. The parameters of SPARsim are estimated by 'SPARSim_estimate_parameter_from_data' function. And then we train the model and generate simulated data through the 'SPARSim_simulation' function.

Matilda. The detailed information of Matilda can be seen in 'Dimension reduction methods' section. Matilda can generate simulated data of two modalities. After the Matilda is trained, we use the function 'get_vae_simulated_data_from_sampling' to generate simulated data. And then we select the simulated data of RNA from the result."

3. Data description of the real datasets including SNARE-seq, PBMC, SHARE-seq and CITE-seq is unclear. For example, the dimension of the SNARE-seq datasets, the evaluation platform for the gene expression or chromatin accessibility from some of the datasets. What does L1, L2, L3 cell type resolutions mean in CITE-seq dataset?

Author's Response: Thanks for your suggestion. In our revised manuscript, we add the description of datasets and cell types in the CITE-seq dataset:

"Description of the dataset

The datasets used in the experiment are mainly matched datasets which contain matched RNA and ATAC/ADT data. There are four datasets used in the experiment:

SNARE-seq dataset. The original RNA and ATAC count matrices are measured from the mouse cerebral cortex by Chen et al [9] and can be downloaded from the GEO website (accession code GSE126074). SNARE-seq contain matched RNA and ATAC data. We follow the processing steps of Lin et al [19] for this dataset and obtain the pre-processed data. It consists of 9190 cells with 241757 features in ATAC and 28930 genes in RNA whit 22 cell types.

PBMC dataset. The 10x-Multiome-Pbmc10k dataset is downloaded from the 10xgenomics [25] to obtain original gene expression and chromatin accessibility. We download this dataset from the preprocessed data provided by Cao et al [44]. It consists of 9631 cells with 107194 features in ATAC and 29095 genes in RNA with 19 cell types.

SHARE-seq dataset. This dataset measures gene expression and chromatin accessibility in the same single-cell in mouse skin samples which is derived from Ma et al. [10]. The raw data is available to download from the GEO website (accession code GSE140203). The gene activity score matrix is obtained by Seurat [26], and cells with less than 1% gene expression are filtered out. It consists of 32231 cells with 340341 features in ATAC and 21478 genes in RNA with 22 cell types.

CITE-seq dataset. The raw data of this dataset is downloaded from the GEO website (accession code GSE164378) and provided by Hao et al [26]. We download a preprocessing file of this dataset provided by Lakkis et al [45] and remove cells labeled as Doublet from the cell type. This dataset consists of 161159 cells with 224 proteins in ATAC and 20729 genes in RNA from eight donors, which is treated as eight batches. And it has three cell type resolutions: L1 (8 types), L2 (30 types) and L3 (57 types). L1, L2 and L3 represent different levels of cell type resolution, L1 represents coarse-grained division of cell types, L2 and L3 represent higher-resolution subpopulation division. We only use L2 (30 types) in our experiment."

References:

[9] Chen S, Lake BB, Zhang K. High-throughput sequencing of the transcriptome and chromatin accessibility in the same cell. Nature biotechnology. 2019;37(12):1452–1457.

[10] Ma S, Zhang B, LaFave LM, Earl AS, Chiang Z, Hu Y, et al. Chromatin potential identified by shared single-cell profiling of RNA and chromatin. Cell. 2020;183(4):1103–1116.

[19] Lin Y, Wu TY, Wan S, Yang JY, Wong WH, Wang YR. scJoint integrates atlas-scale single-cell RNA-seq and ATAC-seq data with transfer learning. Nature biotechnology. 2022;40(5):703–710.

[25] PBMC from a healthy donor - granulocytes removed through cell sorting (10k), Singl e Cell Multiome ATAC + Gene Exp Dataset by Cell Ranger ARC 1.0.0, 10x Genomics; 2 020. Available from: https://support.10xgenomics.com/single-cell-multiome-atac-gex/ datasets/ 1.0.0/pbmc_granulocyte_sorted_10k.

[26] Hao Y, Hao S, Andersen-Nissen E, Mauck WM, Zheng S, Butler A, et al. Integrated analysis of multimodal single-cell data. Cell. 2021;184(13):3573–3587.

[44] Cao ZJ, Gao G. Multi-omics single-cell data integration and regulatory inference with graph-linked embedding. Nature Biotechnology. 2022;40(10):1458–1466.

[45] Lakkis J, Schroeder A, Su K, Lee MY, Bashore AC, Reilly MP, et al. A multi-use deep learning method for CITE-seq and single-cell RNA-seq data integration with cell surface protein prediction and imputation. Nature machine intelligence. 2022;4(11):940–952.

4. Not sure what quantitative metrics are used in Figure 2 e-h for clustering performance evaluation.

Author's Response: Thanks for your suggestion. In our revised manuscript, we add the quantitative metrics description of clustering results in the Figure 2 e-h:

"It can be found that scMoMtF achieves higher AMI, NMI and ARI scores in different datasets (Fig. 2e-h). For example, in PBMC dataset, the AMI, NMI and ARI scores of scMoMtF are 0.847, 0.852, 0.740, which outperforms other methods (MultiVI: 0.743, 0.752, 0.510; scMDC: 0.767, 0.775, 0.660; Matilda: 0.821, 0.827, 0.645). In addition, although the performance of Matilda is close to scMoMtF in the SNARE-seq dataset, scMoMtF performs more stable in the other datasets. These experimental results demonstrate the superior performance of scMoMtF in dimension reduction task for single-cell multi-omics data."

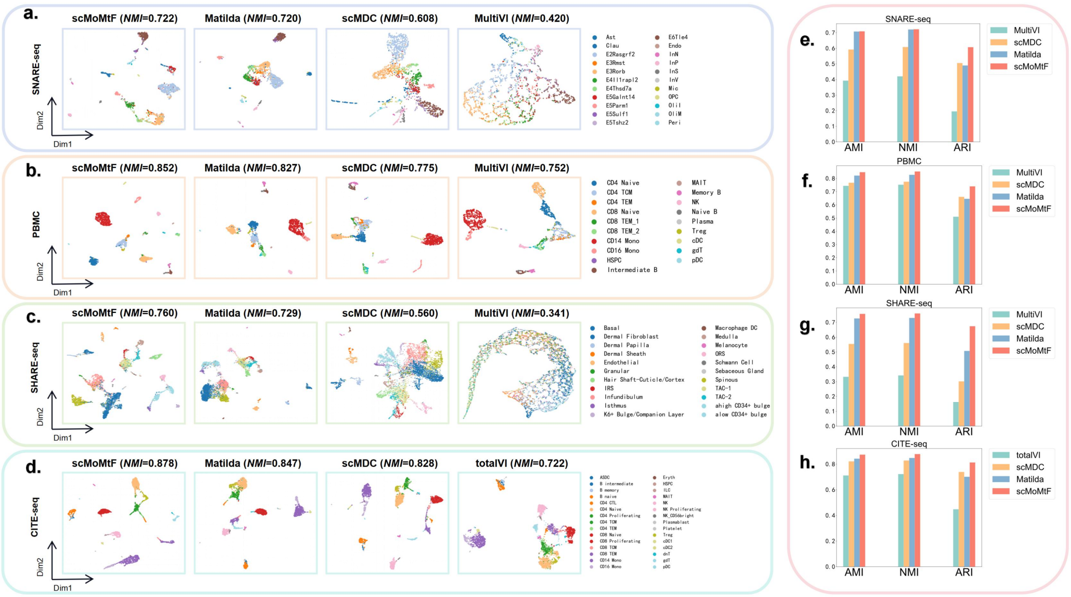

Fig 2. Visualization and performance evaluation of dimension reduction task of scMoMtF compared with other comparison algorithms. a-c Visualization of dimension reduction data generated by scMoMtF, Matilda, scMDC, and MultiVI on SNARE-seq, PBMC, and SHARE-seq datasets. d Visualization of dimension reduction data generated by scMoMtF, Matilda, scMDC and totalVI on the CITE-seq dataset. e-g Evaluate the clustering performance of dimension reduction data generated by scMoMtF, Matilda, scMDC, and MultiVI on SNARE-seq, PBMC, and SHARE-seq datasets using AMI, NMI, and ARI. h The clustering performance of dimension reduction data generated by scMoMtF, Matilda, scMDC and totalVI on CITE-seq dataset.

5. For dimension reduction (Figure 2), how can we tell the developed method is better from Figure 2 a-d? And more details shall be provided in results about the data dimensions after the methods are applied, for example, the proportion of biomarkers that are retained in each omics dataset.

Author's Response: Thanks for your point. In our revised manuscript, we add the analysis of the dimension reduction results to demonstrate the advantages of our method. In addition, we also provide the detail description of the dimension data after dimension reduction and the proportion of biomarkers that are retained in each omics dataset is 50%:

"Performance on single-cell multi-omics data dimension reduction

To evaluate the performance of dimension reduction task on single-cell multi-omics data, we compare scMoMtF with current popular methods including MultiVI [15], totalVI [16], scMDC [17] and Matilda [23]. In these method, MultiVI [15] are designed for RNA modality and ATAC modality. totalVI [16] are designed for RNA modality and ADT modality. scMDC [17] and Matilda [23] are designed for RNA and ATAC/ADT modalities. We set the dimension of the biological information vector of each modality as 150, which is obtained by modality encoder. In addition, the dimension of cell embedding is set to 64 by using cell encoder. For all comparison methods, we use their default dimension for experiments. It should be noted that the donor 2 of CITE dataset and all data of other three datasets are selected as experimental data. We visualize the cell embedding of each model by using uniform manifold approximation and projection (UMAP) (Fig. 2a-d). It can be found the cell embedding of scMoMtF can provide clearer division between different cell clusters, especially for small numbers of cell subtypes. For example, scMoMtF can clearly separate the three cell subtypes (B intermediate, B memory and B naive), while the other methods can not exhibit clear cell cluster boundaries in CITE-seq dataset. In order to intuitively show the dimension reduction performance of each method, we use k-means clustering algorithm to cluster the cell embedding with same parameters (n clusters is the number of cell types for the corresponding dataset and n init is set to 30). We use three quantitative metrics including adjusted mutual information (AMI), normalized mutual information (NMI) and adjusted rand index (ARI) by five-fold cross-validation to measure the cluster performance [29-31]. It can be found that scMoMtF achieves higher AMI, NMI and ARI scores in different datasets (Fig. 2e-h). For example, in PBMC dataset, the AMI, NMI and ARI scores of scMoMtF are 0.847, 0.852, 0.740, which outperforms other methods (MultiVI: 0.743, 0.752, 0.510; scMDC: 0.767, 0.775, 0.660; Matilda: 0.821, 0.827, 0.645). In addition, although the performance of Matilda is close to scMoMtF in the SNARE-seq dataset, scMoMtF performs more stable in the other datasets. These experimental results demonstrate the superior performance of scMoMtF in dimension reduction task for single-cell multi-omics data."

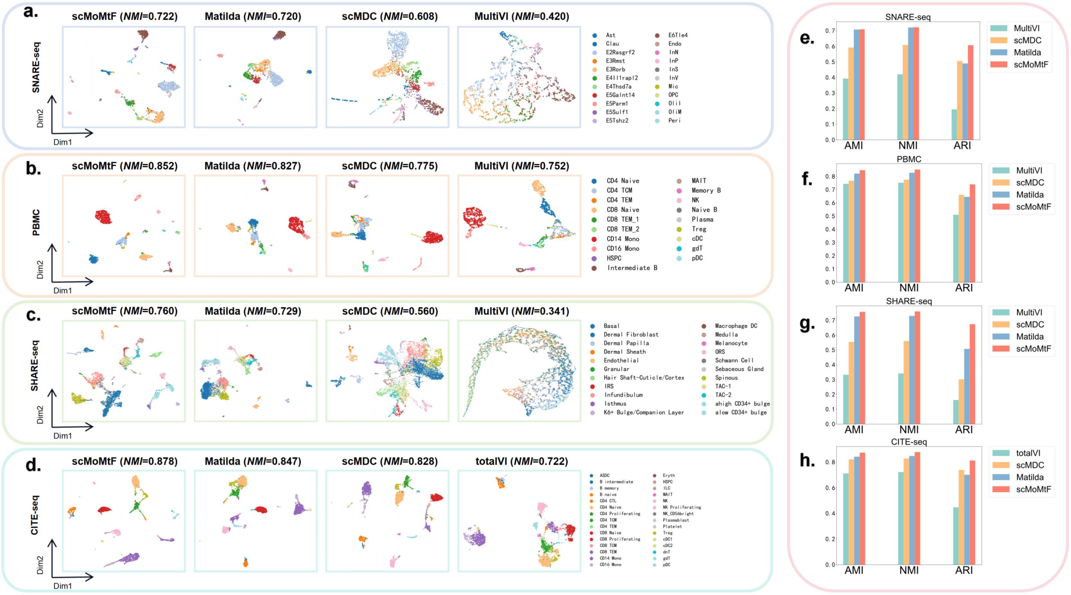

Fig 2. Visualization and performance evaluation of dimension reduction task of scMoMtF compared with other comparison algorithms. a-c Visualization of dimension reduction data generated by scMoMtF, Matilda, scMDC, and MultiVI on SNARE-seq, PBMC, and SHARE-seq datasets. d Visualization of dimension reduction data generated by scMoMtF, Matilda, scMDC and totalVI on the CITE-seq dataset. e-g Evaluate the clustering performance of dimension reduction data generated by scMoMtF, Matilda, scMDC, and MultiVI on SNARE-seq, PBMC, and SHARE-seq datasets using AMI, NMI, and ARI. h The clustering performance of dimension reduction data generated by scMoMtF, Matilda, scMDC and totalVI on CITE-seq dataset.

References:

[15] Ashuach T, Gabitto MI, Koodli RV, Saldi GA, Jordan MI, Yosef N. MultiVI: deep generative model for the integration of multimodal data. Nature Methods. 2023;20(8):1222–1231.

[16] Gayoso A, Steier Z, Lopez R, Regier J, Nazor KL, Streets A, et al. Joint probabilistic modeling of single-cell multi-omic data with totalVI. Nature methods. 2021;18(3):272–282.

[17] Lin X, Tian T, Wei Z, Hakonarson H. Clustering of single-cell multi-omics data with a multimodal deep learning method. Nature communications. 2022;13(1):7705.

[23] Liu C, Huang H, Yang P. Multi-task learning from multimodal single-cell omics with Matilda. Nucleic Acids Research. 2023;51(8):e45–e45.

[29] Zhang Z, Sun H, Mariappan R, Chen X, Chen X, Jain MS, et al. scMoMaT jointly performs single cell mosaic integration and multi-modal bio-marker detection. Nature Communications. 2023;14(1):384.

[30] Meers MP, Llagas G, Janssens DH, Codomo CA, Henikoff S. Multifactorial profiling of epigenetic landscapes at single-cell resolution using MulTI-Tag. Nature Biotechnology. 2023;41(5):708–716.

[31] Lan W, Liu M, Chen J, Ye J, Zheng R, Zhu X, et al. JLONMFSC: Clustering scRNA-seq data

based on joint learning of non-negative matrix factorization and subspace clustering. Methods. 2024;222:1–9.

6. It was not described how the method can simulate cells as mentioned in P6, line 141.

Author's Response: Thanks for your suggestion. Our model can simulate the training data through its generator to augment single-cell multi-omics data. In our revised manuscript, we add the description of data simulation:

"There are two tasks on the single-cell multi-omics data simulation: specific cell type data simulation and multiple cell types data simulation. For specific cell type data simulation task, we apply scMoMtF to the PBMC and CITE-seq datasets. In the PBMC dataset, we use the generator of the trained model to simulate 200 CD14 Mono cells."

7. *Page 11, line 280. What is the decision rule here for determining the real data or fake data?*

Author's Response: Thanks for your point. In our method, the main goal of the discriminator is to distinguish the source of the input data through binary classification. Specifically, the output of discriminator is a probability value that indicates the likelihood that the input data is from real data. We use standard discriminator training methods to achieve this goal by maximizing the discriminator's probability of discriminating real data (close to 1) and minimizing the probability of discriminating fake data (close to 0). Finally, when the output value of the discriminator exceeds 0.5, the data is determined as real data; if it is below 0.5, it is determined as fake data. This is optimized by using cross-entropy loss function to improve the ability of our method to obtain more realistic simulation data.

8. *Page 5, line 129. What does rare cells mean and why this is important?*

Author's Response: Thanks for your point. Regarding the rare cells, we refer to cells that are present in very low abundance within the overall cell population. These rare cells are important in biological and medical research; they can play crucial roles in specific physiological or pathological processes. For example, certain rare cell populations may influence tumor progression or response to therapy in the tumor micro-environment. Additionally, rare subpopulations of immune cells can play key roles in immune responses. Therefore, accurately identifying and analyzing these rare cells is essential for understanding complex biological dynamics. In our revised manuscript, we add the definition of rare cells:

"In addition, it also can be found that scMoMtF is able to correctly classify rare cells in these datasets. For example, comparing with scPred [21] which is the second best model in performance. scMoMtF achieves better classification performance on rare cells (the cell types that have small

proportion in the dataset) such as Plasma (0.1% in the dataset), Treg (1.6% in the dataset) and gdT (1.4% in the dataset) (Fig. 3b-c). The classification accuracy of scMoMtF for Plasma, Treg and gdT is 100%, 80.6% and 85.7%, respectively. The classification accuracy of scPred for Plasma, Treg, and gdT is 66.7%, 77.4% and 53.6%. These results demonstrates that scMoMtF improves the classification accuracy of rare cells which contributes to whole performances improvement of cell classification on different datasets."

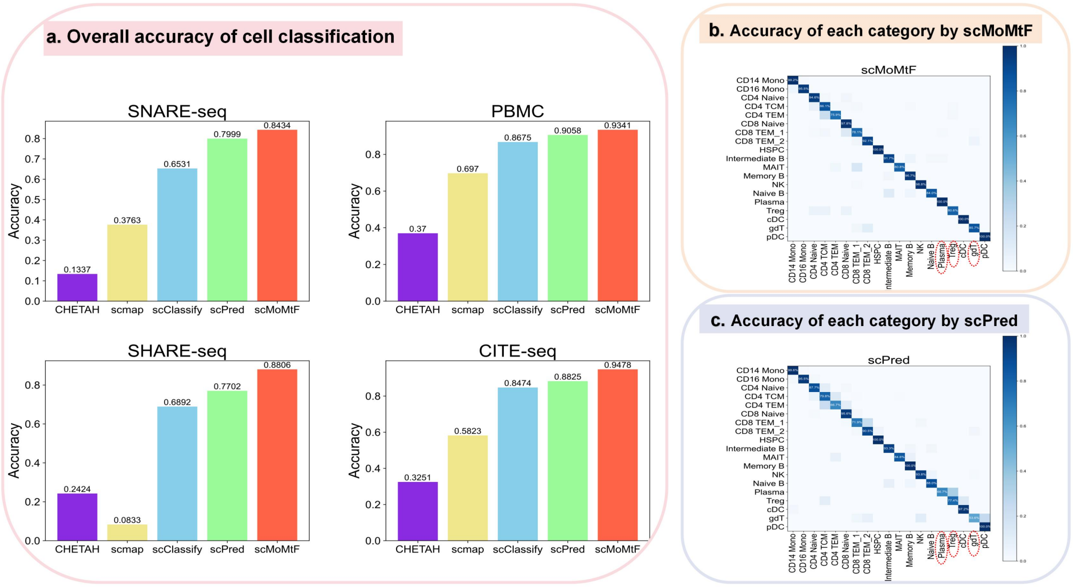

Fig 3. Cell classification performance of scMoMtF. a Comparison of classification accuracy between scMoMtF and other comparison algorithms under five-fold cross-validation. b The classification results of scMoMtF for each cell type in the PBMC dataset. c The classification results of scPred for each cell type in the PBMC dataset.

References:

[21] Alquicira-Hernandez J, Sathe A, Ji HP, Nguyen Q, Powell JE. scPred: accurate supervised method for cell-type classification from single-cell RNA-seq data. Genome biology. 2019;20(1):1–17.

9. *Grammar errors. Just to list a few: Abstract Line 4, comprehensive -comprehensively; Page 3, Line 72, modality-modalities; Page 6, Line 146, selecte-selected; Page 6, Line 158, need-needs; Page 6, Line 164, batche-batches.*

Author's Response: Thanks for your suggestion. In the revised version, we correct these errors and double-check manuscript to correct other errors.
